# Co‐expression of *GR79 EPSPS* and *GAT* yields herbicide‐resistant cotton with low glyphosate residues

**DOI:** 10.1111/pbi.12744

**Published:** 2017-05-26

**Authors:** Chengzhen Liang, Bao Sun, Zhigang Meng, Zhaohong Meng, Yuan Wang, Guoqing Sun, Tao Zhu, Wei Lu, Wei Zhang, Waqas Malik, Min Lin, Rui Zhang, Sandui Guo

**Affiliations:** ^1^ Biotechnology Research Institute Chinese Academy of Agricultural Sciences Beijing China; ^2^ Department of Plant Breeding and Genetics Bahauddin Zakariya University Multan Pakistan

**Keywords:** glyphosate resistant, glyphosate residue, GR79 EPSPS, GAT, cotton

## Abstract

Glyphosate‐resistant (GR) crops have been adopted on a massive scale by North and South American farmers. Currently, about 80% of the 120 million hectares of the global genetically modified (GM) crops are GR crop varieties. However, the adoption of GR plants in China has not occurred at the same pace, owing to several factors including, among other things, labour markets and the residual effects of glyphosate in transgenic plants. Here, we report the co‐expression of codon‐optimized forms of *GR79 EPSPS* and *N‐acetyltransferase* (*GAT*) genes in cotton. We found five times more resistance to glyphosate with 10‐fold reduction in glyphosate residues in two *pGR79 EPSPS*‐*pGAT* co‐expression cotton lines, GGCO2 and GGCO5. The GGCO2 line was used in a hybridization programme to develop new GR cottons. Field trials at five locations during three growing seasons showed that *pGR79*‐*pGAT* transgenic cotton lines have the same agronomic performance as conventional varieties, but were USD 390‐495 cheaper to produce per hectare because of the high cost of conventional weed management practices. Our strategy to pyramid these genes clearly worked and thus offers attractive promise for the engineering and breeding of highly resistant low‐glyphosate‐residue cotton varieties.

## Introduction

Glyphosate is a nonselective, broad‐spectrum, systemic herbicide used for the control of weeds (Beckie, 2011; Heap, 2014; Zhu *et al*., 2000). It competitively inhibits enolpyruvyl‐shikimate‐3‐phosphate synthase (EPSPS), a key plastid‐localized enzyme that functions in the biosynthesis of aromatic amino acids (Duke and Powles, 2008a; Green, 2012). Microbial EPSPS enzyme variants that are not inhibited by glyphosate, particularly CP4 EPSPS*,* have formed the basis of commercially available genetically engineered GR crops (Owen, 2004; Watrud *et al*., 2004). To date, in addition to CP4 EPSPS, several target EPSPS enzymes, including G2 EPSPS (Guo *et al*., 2015), G6 EPSPS (Li *et al*., 2013) and mutant EPSPS (Tian *et al*., 2011), have been successfully cloned and used to achieve glyphosate tolerance in transgenic plants. The pace of adoption of GR crops has been extremely rapid; glyphosate/GR technology has become the foundation of weed management in several crop production systems in the Americas since 1996 (Duke and Powles, 2008b). The high adoption rate of GR technology can be attributed to its obvious benefits to producers, including its simplicity of use, effectiveness in controlling weeds and favourable cost–benefit ratio (Green, 2012). GR crops have been adopted on a massive scale by North and South American producers (Duke and Powles, 2008b). Currently, more than 80% of the 120 million hectares of the global production area of GM crops are planted with GR crop varieties (Busi *et al*., 2013; Dill *et al*., 2008). Consequently, glyphosate is the world's most extensively used herbicide; about 85 million kilograms of glyphosate is applied annually (Beckie, 2011).

The physicochemical properties of the glyphosate molecule enable it to be translocated from the leaf to meristems, young roots, storage organs and other actively growing tissues of plants (Gougler and Geiger, 1981; Pline *et al*., 2002b). This property, along with the growing preponderance of glyphosate in weed management programmes, has raised two nontrivial challenges in agricultural production. First, the translocation and accumulation of glyphosate in plant meristems and reproductive structures can seriously interfere with plant development, can reduce pollen viability and can decrease crop yield (Chen *et al*., 2006; Mei *et al*., 2015; Pline *et al*., 2002a). Second, there are concerns that glyphosate residues may be harmful to human health (Bolognesi *et al*., 2009; De Roos *et al*., 2005; George *et al*., 2010; Greim *et al*., 2015; Guyton *et al*., 2015; Mink *et al*., 2011). Keeping in view the above‐mentioned challenges for GM crop‐based weed control methods, it will be encouraging to introduce the new herbicide‐resistant crop varieties having the ability for less accumulation of herbicide residues.

It has long been established that acetylation, hydrolysis and oxidative cleavage are effective strategies for glyphosate detoxification and residue removal (Castle *et al*., 2004; Guo *et al*., 2015; Pedotti *et al*., 2009; Siehl *et al*., 2005). Several *N*‐acetyltransferases (GAT) have been shown to use glyphosate as a substrate for the production of *N*‐acetylglyphosate via an acetylation reaction. *N*‐Acetylglyphosate is less toxic than glyphosate and is not an effective inhibitor of EPSPS (Castle *et al*., 2004; Liu *et al*., 2015). It can be further metabolized to the nonphytotoxic compound, *N*‐acetyl‐aminomethyl phosphonic acid (*N*‐acetyl‐AMPA) and aminomethyl phosphonic acid (AMPA). *N*‐acetyl‐AMPA and AMPA can be conjugated with natural plant constituents, resultantly less amount of metabolites, or degraded to one‐carbon fragments that are incorporated into natural products (EFSA, 2009). Although plants transgenically expressing *GAT* have significant resistance to glyphosate, GAT and EPSPS enzymes participate in different glyphosate resistance function mechanisms (Guo *et al*., 2015; Liu *et al*., 2015). We previously reported that a novel *N*‐acetyltransferase, GAT, was identified from soil microorganisms isolated from an extremely glyphosate‐polluted site. We found that GAT conferred significantly increased tolerance to glyphosate in *E. coli* (Dun *et al*., 2006), implying that it may be able to confer glyphosate tolerance *in planta*. Recently, we identified a novel class II EPSPS, GR79 EPSP, from the nitrogen‐fixing *Pseudomonas stutzeri* strain A1501; this strain is widely distributed in the wild and occupies diverse ecological niches (Liang *et al*., 2008). Combined with biochemical studies, GR79 EPSPS has high catalytic activity and glyphosate tolerance but a low affinity for glyphosate (Liang *et al*., 2008). Thus, both the *GAT* and *GR79 EPSPS* genes appear to have great potential for use in the genetic engineering of GR plants. These studies prompted us examine whether the co‐expression of *GR EPSPS* and *GAT* genes could be an effective strategy for developing high‐GR, low‐glyphosate‐residue crops.

## Results and discussion

### Preparation of *pGR79 EPSPS* and *pGAT* co‐expression constructions in plant

To facilitate the expression of the *GAT* and *GR79 EPSPS* genes in plants, we designed and synthesized plant‐codon‐optimized versions of *GAT* (designated hereafter as *pGAT*) (Figure S1) and *GR79 EPSPS* (designated hereafter as *pGR79 EPSPS*) (Figure S2). Relative to the wild‐type sequences, these optimized versions of the two genes had higher codon adaptation index values (CAI) (Figure S3a‐d), higher frequencies of optimal codons (FOP) (Figure S3e‐h), and GC content levels predicted to be more suitable for expression in plants (Figure 3i‐l).

**Figure 1:**
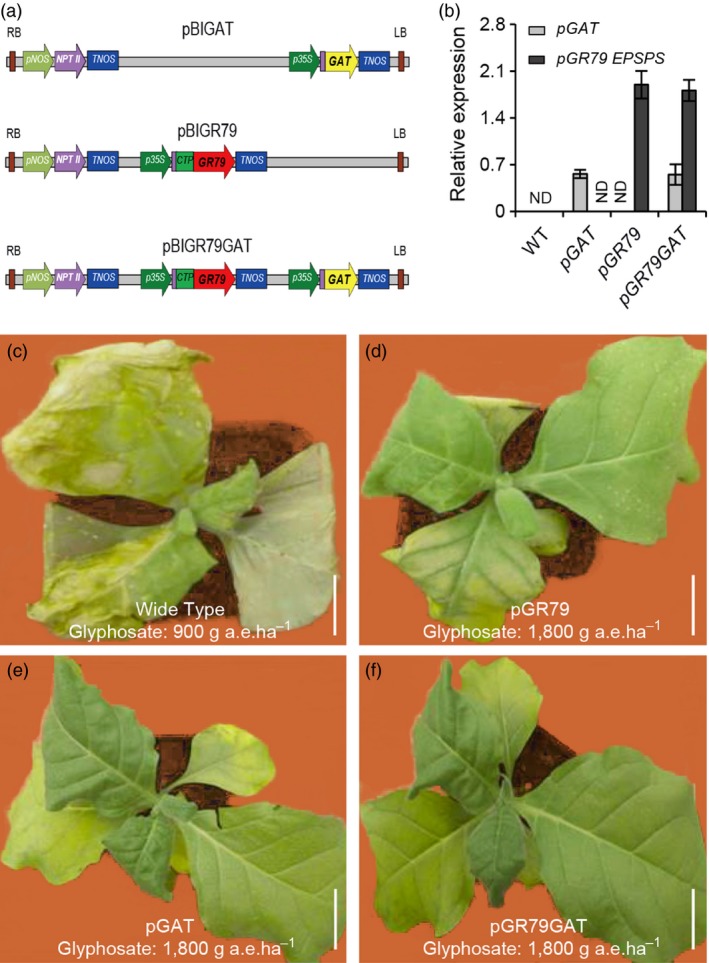
Transgenic tobacco co‐expressing *pGR79 EPSPS* and *pGAT* shows high glyphosate resistance. (a) Vector construction diagram. Purple boxes represent enhancer elements. The pink box represents the *nptII* expression cassette. CTP, chloroplast‐localized signal peptide. (b) RNA levels of *pGR79 EPSPS* and *pGAT* in transgenic tobacco lines. Relative gene expression levels in wild type and in plants individually transformed with *pGR79 EPSPS* or *pGAT* or cotransformed with *pGR79 EPSPS* and *pGAT*. RNA samples used for the assay were prepared using 4‐week‐old seedlings. Independent lines with similar expression levels were used in subsequent analyses. ND: not detectable. (c–f) Wild‐type tobacco was killed at the dose of 900 g a.e./ha glyphosate by 7 day after application (DAA). (d‐f) *pGR79‐pGAT* transgenic tobacco plants (d) showed higher tolerance to the 1800 g a.e./ha glyphosate application than did the *pGR79 EPSPS* (e) or *pGAT* (f) single‐gene tobacco plants. Scale bar, 5 cm.

EPSPS synthase is a chloroplast‐localized enzyme of the shikimate pathway (Watrud *et al*., 2004; Zhu *et al*., 2000). We added a sequence coding for a chloroplast transit peptide in front of the *pGR79 EPSPS* sequence in the transgene cassette (Figure 1a). The chimeric enzyme produced by this fusion was precisely and rapidly guided into chloroplasts and was proteolytically processed to yield an enzyme that conferred stable glyphosate resistance in plants (Owen, 2004). The constitutive *35S* promoter from cauliflower mosaic virus was used to drive the ubiquitous expression of *pGAT* in plant cells; the aim of this ubiquitous expression was to minimize the accumulation of glyphosate residues (Figure 1a).

### Transgenic tobacco co‐expression of pGR79 EPSPS and pGAT enhanced the resistance to glyphosate

First, we demonstrated that co‐expression of *pGAT* and *pGR79 EPSPS* in transgenic tobacco plants resulted in similar levels of *pGAT* and *pGR79 EPSPS* transcription as with single‐gene transformants for these genes (Figure 1b). These *pGAT* and *pGR79 EPSPS* co‐expression tobacco plants were used for subsequent analyses. These plants were grown in a growth room and treated with glyphosate at the following doses: 900 and 1800 g acid equivalents per hectare (a.e./ha). Seven days after the glyphosate application, the entire leaf surface and the shoot apical meristems of the nontransgenic plants were shrivelled and showed severe herbicide‐damage symptoms, while all of the transgenic lines tolerated the 900 g a.e./ha dose (Figure 1c). When the concentration of glyphosate reached 1800 g a.e./ha, the transgenic tobacco plants that contained only *pGAT* or only *pGR79 EPSPS* demonstrated typical damage symptoms for glyphosate application, especially in young leaves. However, the tobacco plants co‐expressing *pGAT* and *pGR79 EPSPS* showed no symptoms, even at the 1800 g a.e./ha of glyphosate dose (Figure 1d–f). These promising observations established the feasibility for the use of *pGAT* and *pGR79 EPSPS* co‐expression as a strategy to develop plants with strong glyphosate resistance.

### Co‐expression of *pGR79 EPSPS* and *pGAT* conferred high resistance to glyphosate in cotton

**Figure 2:**
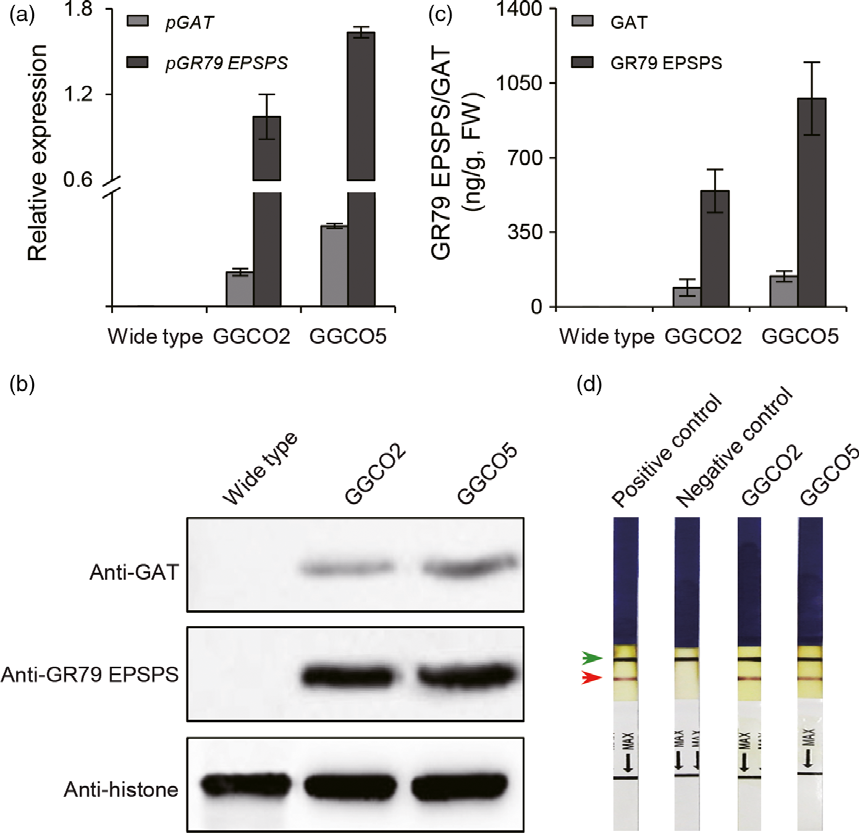
Molecular analyses of transgenic cotton lines co‐expressing *pGR79EPSPS* and *pGAT*. (a) qRT‐PCR analysis of the transgenic expression of *pGR79 EPSPS* and *pGAT* in 6‐week‐old transgenic cotton plants. The cotton *Actin7* gene was used as control to normalize expression levels. (b) Western blot analysis of the expression of GR79 EPSPS and GAT in transgenic cotton lines GGCO2 and GGCO5. (c) Enzyme‐linked immunosorbent assay (ELISA) analysis of GR79 EPSPS and GAT protein expression in the GGCO2 and GGCO5 cotton lines. (d) ImmunoStrip genotyping of GGCO2 and GGCO5 cotton. The red arrow indicates pGR79 EPSPS capture lines. The blue arrow indicates the control line (anti‐mouse lg G). Total protein extractions from 6‐week‐old cotton leaves were analysed by immunoblotting using anti‐GR79 EPSPS antibodies.

**Figure 3:**
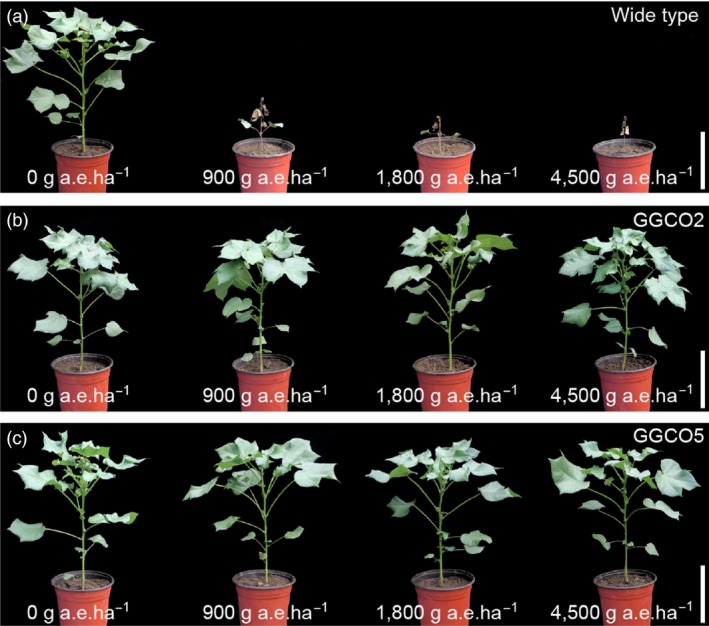
Transgenic cotton plants co‐expressing *pGR79 EPSPS* and *pGAT* are resistant to high glyphosate doses. (a) Wild‐type cotton sprayed with glyphosate at the following doses: 0 g a.e./ha, 900 g a.e./ha, 1800 g a.e./ha and 4500 g a.e./ha. Scale bar, 10 cm. (b) GGCO2 cotton was sprayed with glyphosate at the following doses: 0 g a.e./ha, 900 g a.e./ha, 1800 g a.e./ha and 4500 g a.e./ha. Scale bar, 10 cm. (c) GGCO5 cotton was sprayed with glyphosate at the following doses: 0 g a.e./ha, 900 g a.e./ha, 1800 g a.e./ha and 4500 g a.e./ha. Scale bar, 10 cm.

We next introduced the *pGAT* and *pGR79 EPSPS* co‐expression cassette into upland cotton variety R18 (*Gossypium hirsutum*). Quantitative real‐time (qRT)‐PCR was used to screen for transgenic lines that had high expression levels for both *pGAT* and *pRG79 EPSPS*. Seven independent transgenic lines were ultimately selected based on careful monitoring of the expression levels of both transgenes over several generations (Figures 2a and S4). The GR traits of the transgenic lines carrying were assessed using homozygous GGCO2 (harbouring a single copy of the *pGAT‐pRG79 EPSPS* cassette) and GGCO5 (harbouring two copies of the *pGAT‐pRG79 EPSPS* cassette) plants from the T_5_ generation (Figure S5). GGCO2 and GGCO5 transgenic cotton plants were examined through Western blotting (Figure 2b) and enzyme‐linked immunosorbent assays (ELISAs) (Figure 2c). These analyses confirmed the up‐regulation of both GR79 EPSPS and GAT. Further, we developed a method based on ImmunoStrip analysis to simply and rapidly detect the presence of the GR79 EPSPS proteins in the GGCO2 and GGCO5 cotton lines (Figure 2d). The GGCO2 and GGCO5 plants had significant increases in GR as compared to transgenic cotton plants expressing only *pGAT* or only *pGR79 EPSPS* (Figure 3). Moreover, the GGCO2 and GGCO5 plants remained green and vigorous even following 1800 and 4500 g a.e./ha applications of glyphosate (Figure 3). This impressive GR performance clearly demonstrates effectiveness and practical utility of this co‐expression GR strategy in cotton.

### *pGR79 EPSPS‐pGAT* co‐expression cottons showed 10‐fold reduction in glyphosate residues

**Figure 4:**
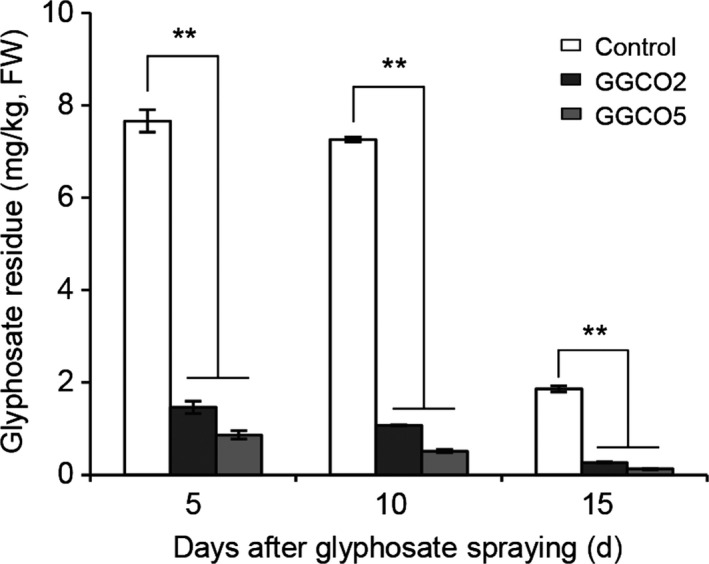
Transgenic cotton plants co‐expressing *pGR79 EPSPS* and *pGAT* exhibit significantly reduced glyphosate‐residue levels compared with other transgenic GR genotypes. Glyphosate‐residue levels in GGCO2 and GGCO5 cotton leaves after glyphosate applications. FW, fresh weight. **P ≤ 0.01; Student's *t*‐test.

Like glyphosate, *N*‐acetylglyphosate is chemically stable and is not metabolized by plants. Our study clearly demonstrated that the cotton plants were morphologically normal following the exogenous application of *N*‐acetylgyphosate for 4 weeks (Figure S6). However, this chemical is not an herbicide, and it has no known toxicity in humans or animals (Castle *et al*., 2004; EFSA, 2009; Siehl *et al*., 2005). To examine whether the GAT enzyme that catalyses the acetylation of secondary amines of glyphosate could metabolize glyphosate in transgenic cotton, the total glyphosate content in leaves of GGCO2 and GGCO5 plants was measured. Compared to *pGR79 EPSPS* single‐gene transformant plants, the co‐expression lines had a rapid and significant decrease in glyphosate‐residue levels in the leaves 5 day after an application of 900 g a.e./ha glyphosate (Figure 4). The glyphosate concentrations in the GGCO2 and GGCO5 plants were 80.9% (1.46 PPM) and 88.7% (0.87 PPM), respectively, lower than that of the *pGR79 EPSPS* single‐gene plants. At 10 day postapplication, the GGCO2 and GGCO5 plants had 85.2% and 92.9% reductions in glyphosate compared to plants expressing only *pGR79 EPSPS*. Similarly, at 15 day postapplication, the co‐expression plants had 85.7% (GGCO2) and 93.2% (GGCO5) reductions in glyphosate content relative to the *pGR79 EPSPS‐*only transgenic plants (Figure 4). These impressive reductions in glyphosate content may result from glyphosate acetylation as catalysed by GAT (Castle *et al*., 2004).

### Field evaluation of *pGR79 EPSPS‐pGAT* cotton and new *pGR79 EPSPS‐pGAT* varieties

After observing similar GR performance in both the GGCO2 (single copy) and GGCO5 (two copy) lines, the transgenic line with single‐copy GGCO2 was used for hybridization. Prior to hybridization, flanking sequence analysis revealed that the GGCO2 insertion site is an intergenic region of Chromosome D10 (nucleotide 20 274 741 to 20 274 752) (Figure S7). Therefore, the observed GR in the GGCO2 cotton line can be concluded to be directly related to the activity of the introduced *GAT* and *GR79 EPSPS* genes, rather than because of the physical insertion site of the transgenic construct at a particular position in the cotton genome. Importantly, there were no significant differences in agronomic performance between the newly developed GR cotton cultivars and their respective parental genotypes (Figure S8).

**Figure 5:**
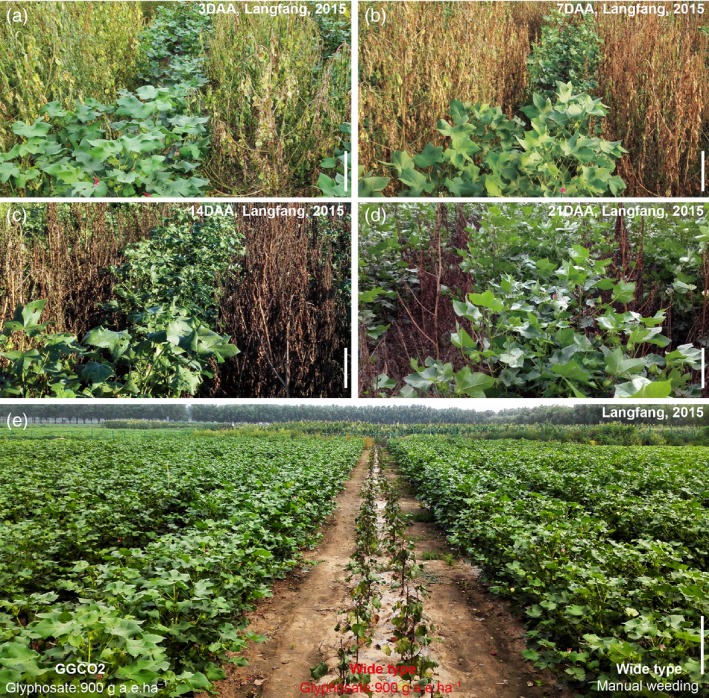
Field evaluations of GGCO2 and the new *pGR79*‐*pGAT* cotton varieties. (a–d) Pictures of GGCO2 transgenic line 3 days (a), 7 days (b), 14 days (c), and 21 days (d) after a 900 g a.e./ha application of glyphosate on an experimental farm in Langfang (Hebei province). (e) A field view showing the phenotypes of control and GGCO2 cotton plants in Langfang (Hebei province), 2013.

Field evaluations of GGCO2 plants were conducted in three planting seasons at five different geographical locations across China: Beijing (N39°54′, E116°24′), Langfang (Hebei province, N39°56′, E116°20′), Macheng (Hubei province, N31°08′, E114°57′), Dongying (Shandong province, N37°25′, E118°40′) and Sanya (Hainan province, N18°15′, E109°30′). At the four‐leaf stage, 900 g a.e./ha of glyphosate was applied to GGCO2 plants, nontransgenic cotton plants, and the various weed species present in the local production systems of the various trial sites. We observed the rapid appearance of chlorosis, necrosis and wilting that led to the death of all of these plants with the exception of the GGCO2 plants (Figure 5a–e).

Hybridization between the GGCO2 line and various glyphosate‐susceptible genotypes, including 3 non‐*Bacillus thuringiensis* (Bt) and 16 Bt cotton varieties from Asian countries (China, India, Pakistan and Australia), was performed to introduce glyphosate resistance into popular genetic backgrounds (Figure S9a and Table S1). ImmunoStrip analysis was used to confirm the presence of the GR79 EPSPS proteins in the newly developed GR cotton lines (Figure S9b). Similar to GGCO2, all 19 of the new cultivars with the *pGAT‐pRG79 EPSPS* co‐expression cassette were highly resistant to glyphosate and had low glyphosate‐residue levels under field conditions. For the new cotton cultivars, the required number of glyphosate applications varied among the field trial location sites, with a minimum of two and a maximum of four applications needed to prevent yield losses caused by weeds. The two applications of glyphosate cost approximately $105/ha; the four applications cost $210/ha. Of note, conventional weed eradication from cotton production fields is labour intensive and expensive; the measures required to prevent yield loss by weeds for the non‐GR cotton cultivars cost approximately $600/ha.

## Conclusions

In summary, the present study demonstrated that pyramiding of glyphosate resistance and detoxification genes conferred highly glyphosate‐resistant plants that had low glyphosate‐residue levels and did not suffer any yield losses. Although the exact biochemical mechanism has not been verified, it seems highly likely that the introduction of the detoxification *GAT* gene rapidly degraded significant amounts of glyphosate residues, resulting in more robust of glyphosate, thereby allowing the frequent use of glyphosate for weed control in farmer field. In the past decade, the planting area in China devoted to cotton production has dropped by around one third (http://www.stats.gov.cn/), due in part to sharp rises in labour costs. We anticipate that the introduction of *pGR79 EPSPS*‐*pGAT* co‐expression cotton varieties helps to improve the mechanization of weed control in Asian cotton production systems. This strategy will cost less than the conventional weed management methods and promises to increase the economic gains of cotton farmers.

## Experimental procedures

### Plant materials and growth conditions

Tobacco accession NC89 and its derived transgenic plants were grown in an artificial growth chamber at 50% humidity, with a 12‐h light (28 °C)/12‐h dark (25 °C) photoperiod and a photon density of approximately 400 μmol photons/m^2^/s. The tobacco seeds (T_2_) collected from positive T_1_ lines and transgenic homozygous plants were selected based on PCR screening results for the *pGR79 EPSPS*,* pGAT*, and kanamycin‐resistant genes. Cotton variety R18 and its derived transgenic plants were grown in a glasshouses and in the fields of different experimental stations, including Beijing (N39°54′, E116°24′), Langfang (Hebei province, N39°56′, E116°20′), Macheng (Hubei province, N31°08′, E114°57′), and Dongying (Shandong province, N37°25′, E118°40′), from April to October, and in Sanya (Hainan province, N18°15′, E109°30′) from November to March. Cotton seeds (T_2_) harvested from positive T_1_ lines and transgenic homozygous plants were selected based on PCR screening. The transgenic homozygous lines were selected and self‐fertilized from the T_3_ to the T_5_ generation, until the agronomic traits were stabilized.

### Gene codon optimization

The OptimumGene™ algorithm optimizes various parameters that are critical to the efficiency of gene expression in plants, including codon usage bias, GC content, CpG dinucleotide content, mRNA secondary structures, cryptic splicing sites, premature PolyA sites, internal Chi sites, ribosomal binding sites, negative CpG islands, RNA instability motifs, and repeat sequences. Both of the bacterial genes in this study contain tandem codons that are rare in plants and can therefore reduce the efficiency of translation, or even disengage a transcript from translational machinery. The codon usage bias of the *GAT* and *GR79 EPSPS* genes from *Gossypium* was changed by optimizing the codon adaptation index values (CAI) from 0.91 to 0.86 and from 0.60 to 0.86, respectively. GC content and unfavourable peaks were optimized to prolong the half‐life of the mRNA molecules. Stem‐loop structures, which are known to impact ribosomal binding and the stability of mRNA, were broken. Additionally, the optimization process screened for and successfully modified all of the negative cis‐acting sites present in these genes. This analysis was performed by GenScript (GenScript (Nanjing) Co., Ltd., China).

### Overexpression transgene constructs

The optimal CDS of the *pGAT* (441 bp) and *pGR79 EPSPS* (1338 bp) genes were commercially synthesized (GenScript (Nanjing) Co., Ltd., China) and cloned, both individually and jointly, into the binary vector pBI121‐CaMV 35S to generate vectors overexpressing *pGAT*,* pGR79 EPSPS,* and *pGAT*‐*pGR79 EPSPS*. The resulting vectors were introduced into tobacco variety NC89 via *Agrobacterium*‐mediated transformation. The vector co‐expressing the *pGAT* and *pGR79 EPSPS* genes was transformed into cotton variety R18 via *Agrobacterium*‐mediated transformation to generate plants for the glyphosate resistance analysis.

### RNA extraction, cDNA preparation, and qRT‐PCR

Total RNA was extracted using an RNAprep pure Tissue Kit (TIANGEN, China). Approximately 2 μg of the total RNA was used as a template to generate cDNA with ReverTra Ace^®^ qPCR RT Master Mix with a gDNA removal Kit (Toyobo, Japan). For qRT‐PCR, SYBR Green I was added to the reaction mix; the analysis was performed with a Chromo4 real‐time PCR detection system according to the manufacturer's instructions (Bio‐Rad). Expression data were analysed with Opticon monitor software (Bio‐Rad). *NtActin9* and *GhActin7* were used as internal controls in the tobacco and cotton analyses, respectively. The primers used for PCR and qRT‐PCR are listed in Table S2.

### *Agrobacterium*‐mediated genetic transformation and regeneration of cotton

Transgenic cotton plants were produced according to the method described by Meng *et al*. (2007). Hypocotyl segments taken from *in vitro* grown 6‐day‐old seedlings of cotton cultivar R18 were used as explants for transformation using the GV3101 strain of *Agrobacterium tumefaciens* harbouring pBI121‐P35‐GR79‐P35S‐GAT plasmid. All of the transgenic calli and plants were selected and developed on solid media containing 100 mg/L glyphosate (Sigma, P9556). The transgenic plants were grown in a glasshouse and in the fields of different experimental stations, including Beijing (N39°54′, E116°24′), Langfang (Hebei province, N39°56′, E116°20′), Macheng (Hubei province, N31°08′, E114°57′), and Dongying (Shandong province).

### Southern hybridization analysis

Fresh cotton leaves were used to extract total genomic DNA (40–60 μg) according to previously reported methods (Zhang *et al*., 2015). gDNA was digested with *Eco* RI and *Hind* III (Promega, USA), followed by separation on 0.8% agarose gels. DNA was then transferred to 10 × SSC Hybond N+ nylon membranes via a vacuum‐transfer apparatus (Vacuum Blotter 785; Bio‐Rad, USA), and cross‐linked using UV (CL‐1000 UV Crosslinker; UVP, USA) for 1 min (0.1 J/cm^2^). Southern hybridization was carried out as described previously (Zhang *et al*., 2015). The probes for *pGAT* and *pGR79 EPSPS* were, respectively, the 415‐bp and 786‐bp PCR fragments amplified with the primers listed in Table S2.

### ImmunoStrip analysis

A His‐fused polypeptide was expressed in BMRosetta (DE3) cells. The purified fusion protein was injected into rats to produce polyclonal antibodies against GR79 EPSPS (Beijing Protein Innovation, China). The loading control used was the anti‐HSP82 antibody (Beijing Protein Innovation, China). The ImmunoStrips used in this experiment were manufactured by Beijing Protein Innovation (China). About 500‐mg leaf samples were harvested and homogenized using a tissue grinder. The homogenized samples were incubated for 5 min at room temperature to enable dissociation of the protein complexes and then centrifuged at 12 000 ***g*** for 10 min. ImmunoStrips were then placed into the aqueous‐phase supernatant of the samples and incubated at room temperature for 20 min. Negative controls were performed using the same procedure with the nontransgenic cotton plants.

### Glyphosate tolerance analysis in tobacco and cotton

The glyphosate resistance levels of transgenic tobacco and cotton plants were assessed with commercially formulated isopropylamine salt of glyphosate at a 300 g a.e./L application rate (Roundup, Monsanto Company, USA). The labelled dose for glyphosate application in the GR transgenic tobaccos and cottons in the glasshouse or field was 900, 1800 and 4500 g a.e./ha. The recommended dose for plant production is 900 g a.e./ha according to the manufacturer's manual. Glyphosate resistance analysis of transgenic GR plants was performed according to previously reported methods (Guo *et al*., 2015; Li *et al*., 2013). Transgenic tobaccos were sprayed at the four‐leaf stage with glyphosate doses of 900 and 1800 g a.e./ha. Transgenic cottons were sprayed at the four‐leaf stage with glyphosate doses of 900, 1800 and 4500 g a.e./ha. The phytotoxicity symptoms of GR plants were investigated over the subsequent 4 weeks.

### Glyphosate residues measured by mass spectrometry

The glyphosate residues were extracted and measured from similar leaves at the same position of three independent biological repeats, as described previously (Zhou *et al*., 2008). The cotton leaves were ground in liquid nitrogen, and the homogeneous leaf powder samples (200 mg) were extracted with 10 mL of an aqueous solution containing 0.05% (V/V) trichloroacetic acid as an internal standard. Purification was performed with an Oasis Max solid‐phase extraction cartridge (150 mg/6 cc; Waters) and eluted with methanol, followed with ultrapure water. Analyte derivatization was performed in a new centrifuge tube with 26 μM/L sodium tetraborate and 770 μM/L FMOC‐Cl for 1 h at room temperature. The samples were then injected into a liquid chromatography–tandem mass spectrometry system consisting of an Acquity ultra‐performance liquid chromatograph (Acquity UPLC; Waters) coupled to a triple quadrupole tandem mass spectrometer (Quattro Premier XE; Waters).

### Agronomic trait analysis

Important agronomic traits, including plant height, number of branches, number of bolls per plant, unginned cotton yield per plant, lint cotton yield per plant, boll weight, lint cotton yield per boll, lint percentage, unginned cotton yield per plot and lint cotton yield per plot, were measured on a single‐plant basis.

## Competing financial interests

The authors declare no competing financial interests.

## Supporting information


**Figure S1.** GAT sequence analysis before and after codon optimization.
**Figure S2.** Sequence analysis of pGR79 EPSPS before and after codon optimization.
**Figure S3.** Codon optimization analysis of *pGR79 EPSPS* and *pGAT*.
**Figure S4.** Screening of *pGR79 EPSPS* and *pGAT* co‐expressing cotton for glyphosate tolerance in the T_0_ generation.
**Figure S5.** Generation of *pGR79 EPSPS* and *pGAT* overexpressing transgenic cotton plant lines.
**Figure S6.** Cotton plants were morphologically normal following the exogenous application of *N*‐acetylgyphosate.
**Figure S7.** Identification of the flanking sequence in GGCO2 cotton.
**Figure S8.** Agronomic traits of GGCO2 plants in normal field conditions in Langfang, Hebei province in 2015.
**Figure S9.** ImmunoStrip analysis of the *pGR79 EPSPS*‐*pGAT* cotton varieties.
**Table S1.** GR varieties developed from hybridizations with GGCO2.
**Table S2.** Primers used in this study.
