# Supplementary material for: Co‐expression of GR79 EPSPS and GAT yields herbicide‐resistant cotton with low glyphosate residues
Source: Plant Biotechnol J. 2017 May 26;15(12):1622–9. doi: 10.1111/pbi.12744 (PMC5698046; doi:10.1111/pbi.12744)
Supplement: Supplementary file 1 — Figure S1. GAT sequence analysis before and after codon optimization. Figure S2. Sequence analysis of pGR79 EPSPS before and after codon optimization. Figure S3. Codon optimization analysis of pGR79 EPSPS and pGAT. Figure S4. Screening of pGR79 EPSPS and pGAT co‐expressing cotton for glyphosate tolerance in the T0 generation. Figure S5. Generation of pGR79 EPSPS and pGAT overexpressing transgenic cotton plant lines. Figure S6. Cotton plants were morphologically normal following the exogenous application of N‐acetylgyphosate. Figure S7. Identification of the flanking sequence in GGCO2 cotton. Figure S8. Agronomic traits of GGCO2 plants in normal field conditions in Langfang, Hebei province in 2015. Figure S9. ImmunoStrip analysis of the pGR79 EPSPS‐pGAT cotton varieties. Table S1. GR varieties developed from hybridizations with GGCO2. Table S2. Primers used in this study. [file PBI-15-1622-s001.pdf]

Supplementary figure legends

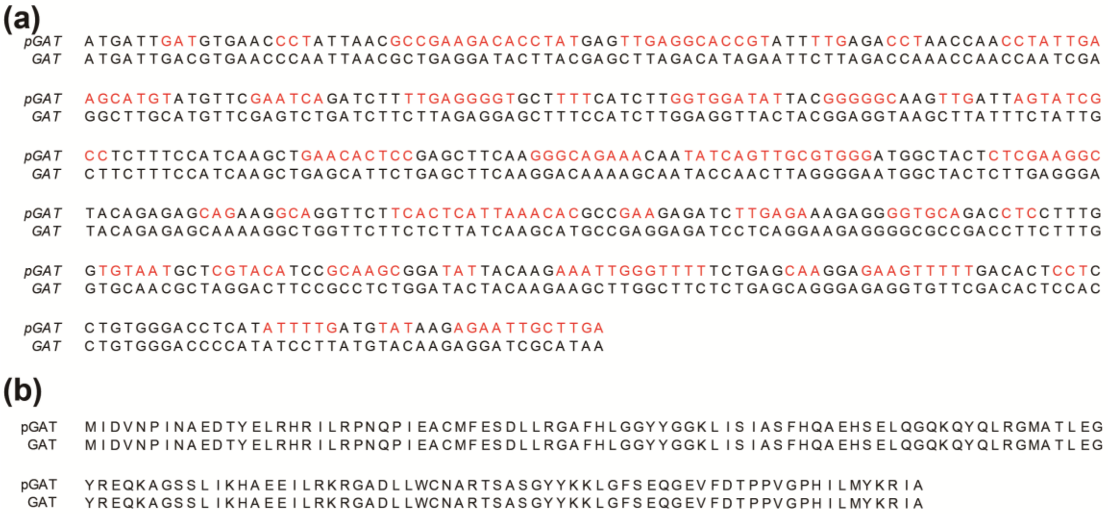

Figure S1 GAT sequence analysis before and after codon optimization.

(**a**) Alignment of the nucleotide sequence of *pGAT* with *GAT*. The red letters indicate the plant codon-optimized sequences.

(**b**) Alignment of the protein sequences of pGAT and GAT.

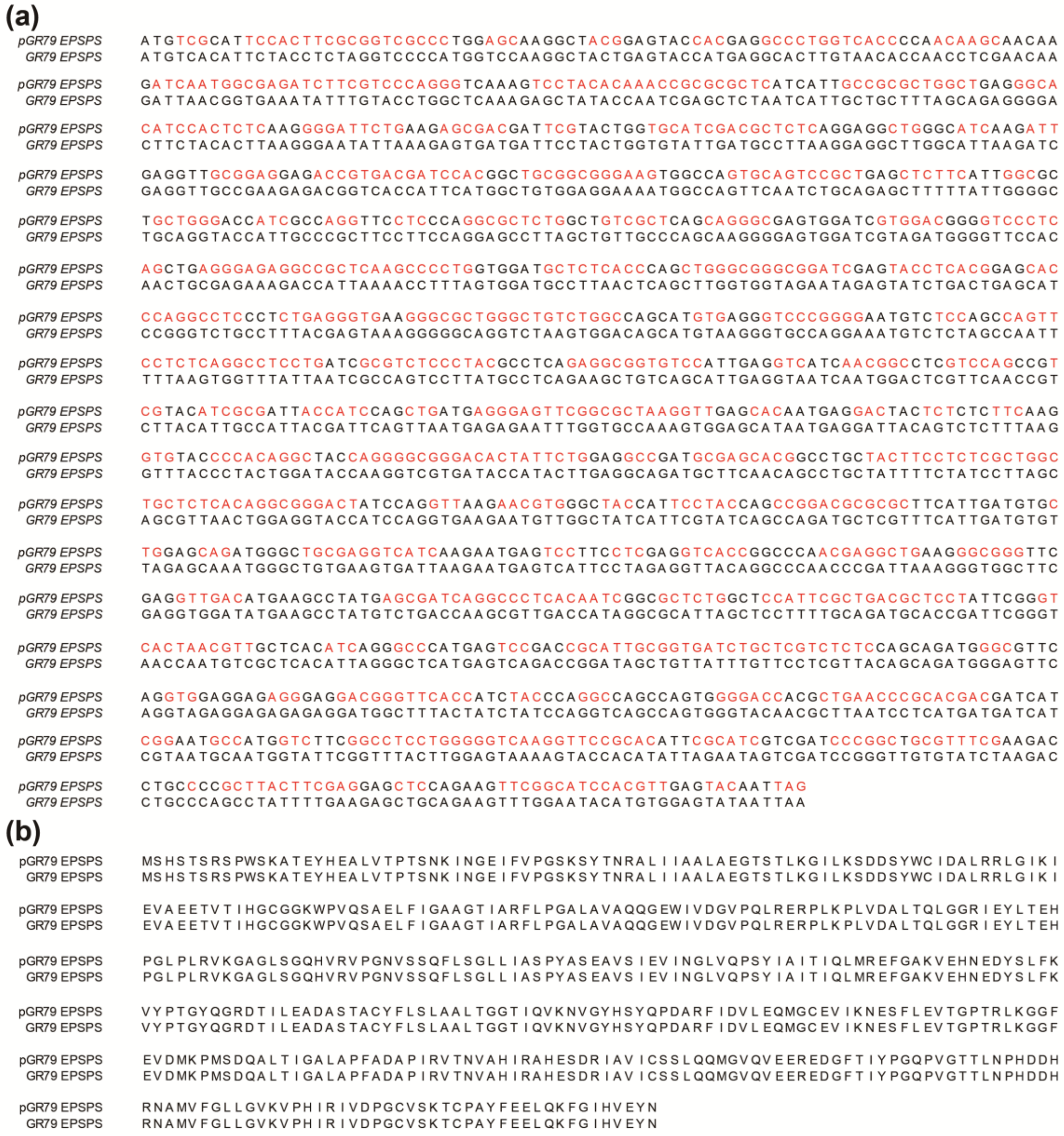

Figure S2 Sequence analysis of pGR79 EPSPS before and after codon optimization.

(**a**) Alignment of the nucleotide sequence of *pGR79 EPSPS* with *GR79 EPSPS*. The red letters indicate the plant codon-optimized sequences.

(**b**) Alignment of the protein sequences of pGR79 EPSPS and GR79 EPSPS.

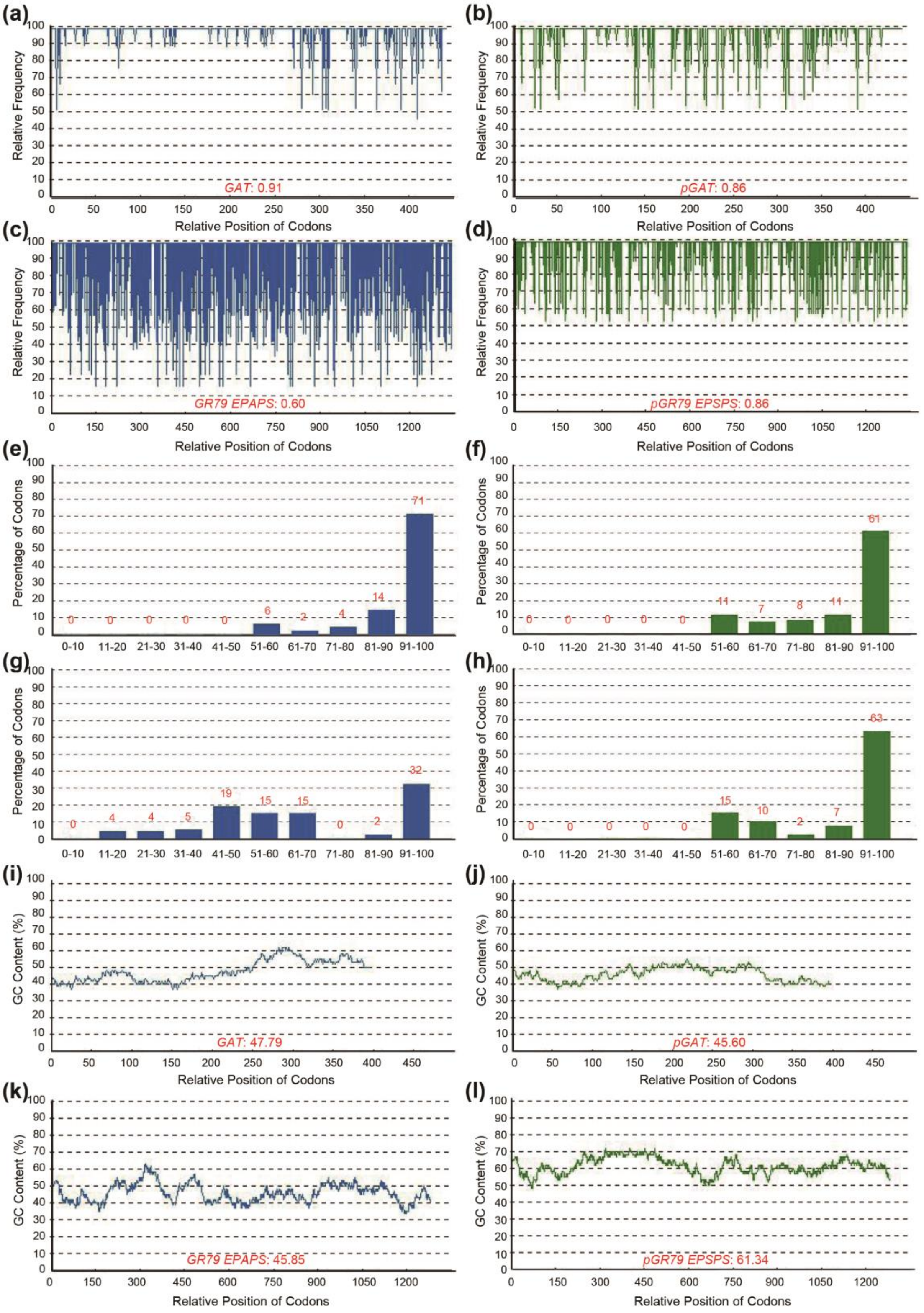

Figure S3 Codon optimization analysis of *pGR79 EPSPS* and *pGAT*.

(**a-h**) Codon usage bias adjustment. (a-d) The distribution of codon usage frequency along the length of the *pGAT* (a and b) and *pGR79 EPSPS* (c and d) sequences. A codon adaptation index (CAI) of 1.0 is considered to be perfect in the desired expression

organism, and a CAI of > 0.9 is regarded as very good, likely indicating a high gene expression level. Blue lines indicate the sequence prior to the OptimumGene™ optimization of CAI. Green lines indicate the sequence following the OptimumGene™ optimization of CAI. (e-h) The percentage distribution of codons in computed codon quality groups. A value of 100 is set for the codon with the highest usage frequency for a given amino acid in the desired expression organism. Blue histograms indicate the groups before OptimumGene™ optimization of frequency of optimal codons (FOP). Green histograms indicate the groups after OptimumGene™ optimization of FOP.

(**i-l**) GC content adjustment. The suitable percentage range of GC content is between 30-70%. Peaks of GC content in a 60bp window have been removed in the pGAT and PGR79 EPSPS genes. Blue wavy lines indicate before OptimumGene™ optimization of GC content. Green wavy lines indicate after OptimumGene™ optimization of GC content.

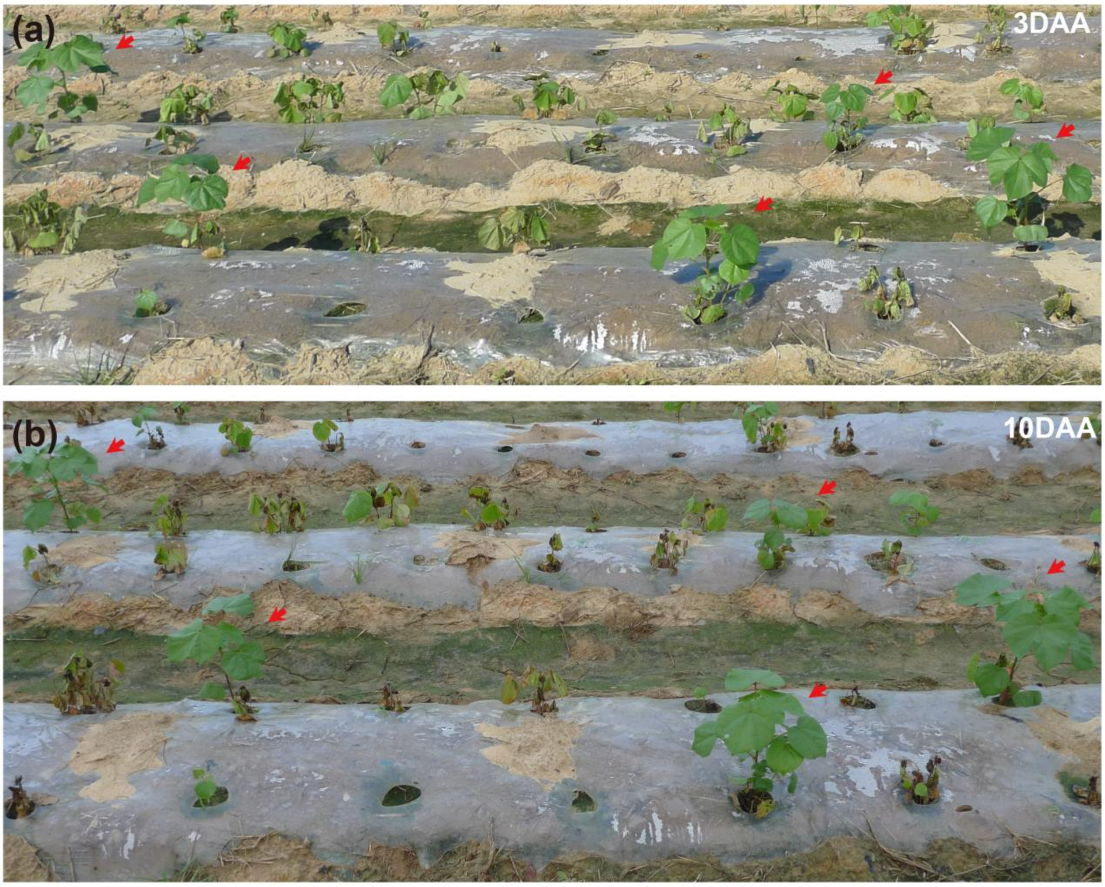

Figure S4 Screening of *pGR79 EPSPS* and *pGAT* co-expressing cotton for glyphosate tolerance in the T_0 generation. Phenotype of 4-week-old transgenic cotton plants sprayed with 900 g a.e.ha^{-1} glyphosate at day 3 (**a**) and day 6 (**b**) post application.

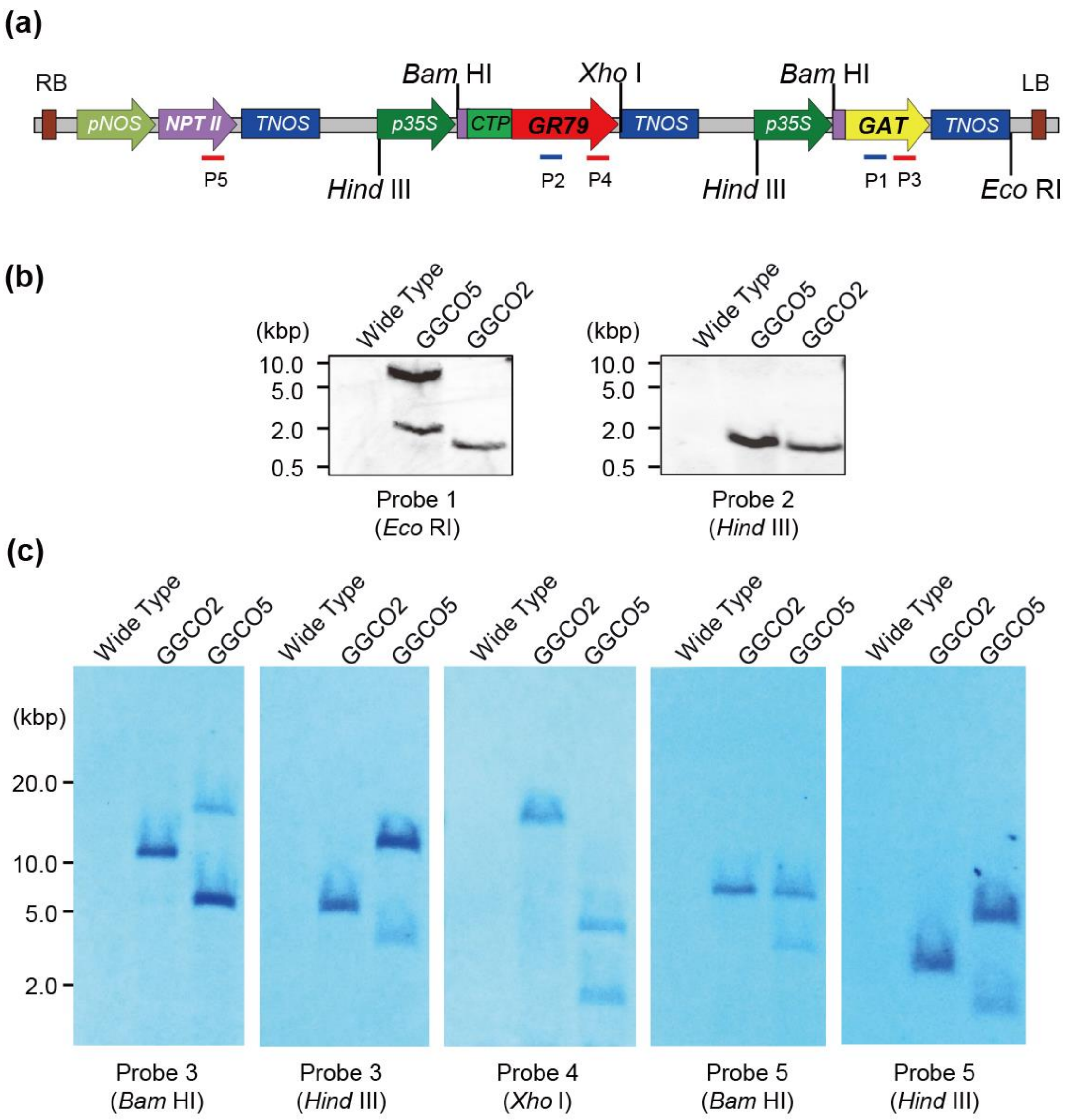

Figure S5 Generation of *pGR79 EPSPS* and *pGAT* overexpressing transgenic cotton plant lines. (a) Schematic representation of the *pGR79GAT* construct, indicating the position of the restriction enzyme sites, including *Hind* III, *Bam* HI, *Xho* I, and *Eco* RI. P1 to P5 indicate the locations of probes 1– to 5. (**b and c**) Screening of transgenic cotton plants with targeted insertions using Southern blotting. (b) Genomic DNA was digested by *Eco*RI and *Hind*III, the targeted insertions were recognized by probe 1 and probe 2 (indicated in (a) with blue boxes). (b), Genomic DNA was digested by *Bam*HI, *Hind*III, and *Xho*I, the targeted insertions were recognized by probes 3-5 (indicated in (a) with red boxes).

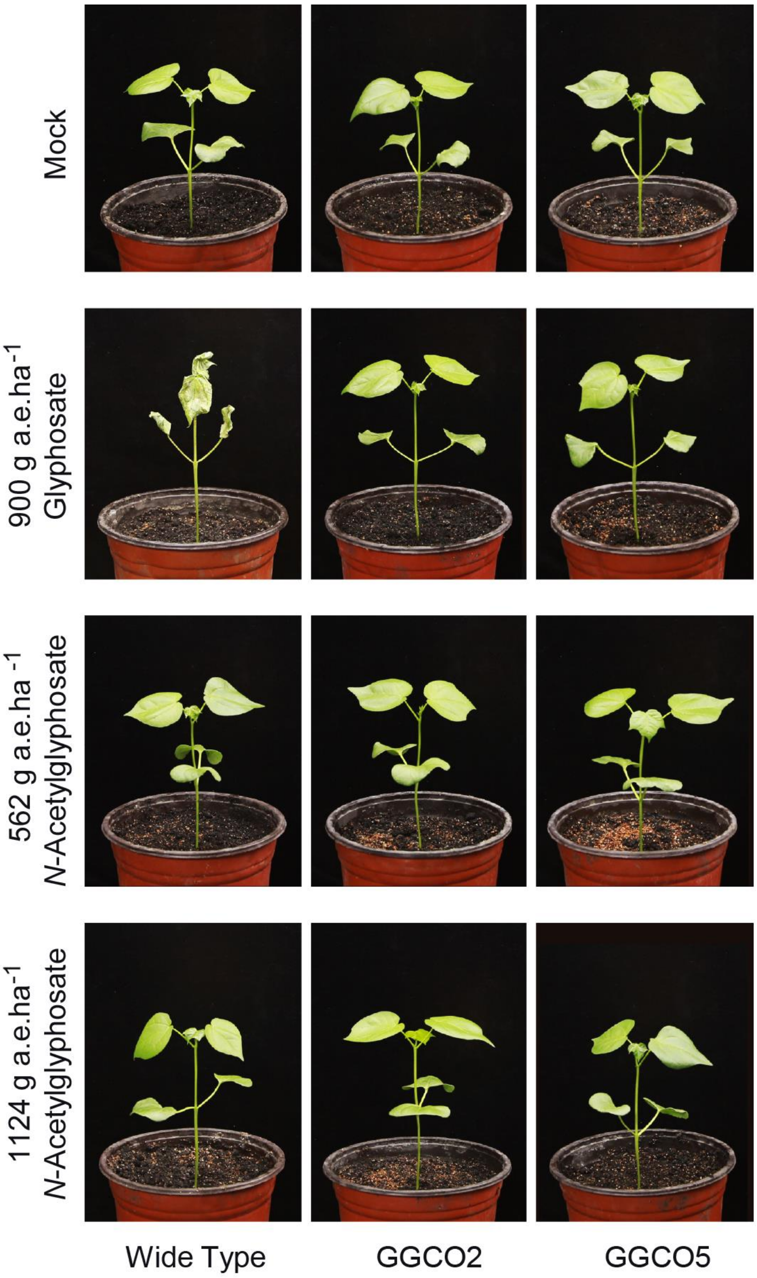

Figure S6 Cotton plants were morphologically normal with exogenous application of *N*-acetylgyphosate.

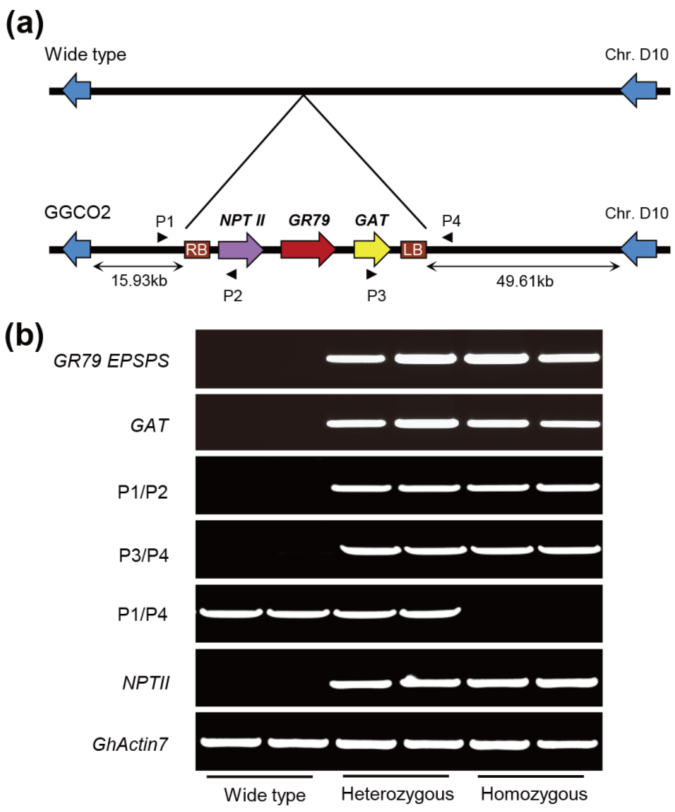

Figure S7 Identification of the flanking sequence in GGCO2 cotton. (**a**) Schematic representation of the *pGR79GAT* vector flanking region in the GGCO2 genotype. LB and RB represent the left and right borders of the pGR79GAT vector, respectively. The red arrows indicate the *GR79 EPSPS* expression cassette. The yellow arrows indicate the *GAT* expression cassette. The pink arrow indicates the *NPTII* expression cassette. (**b**) Flanking sequence was confirmed by PCR using the primers given in (a).

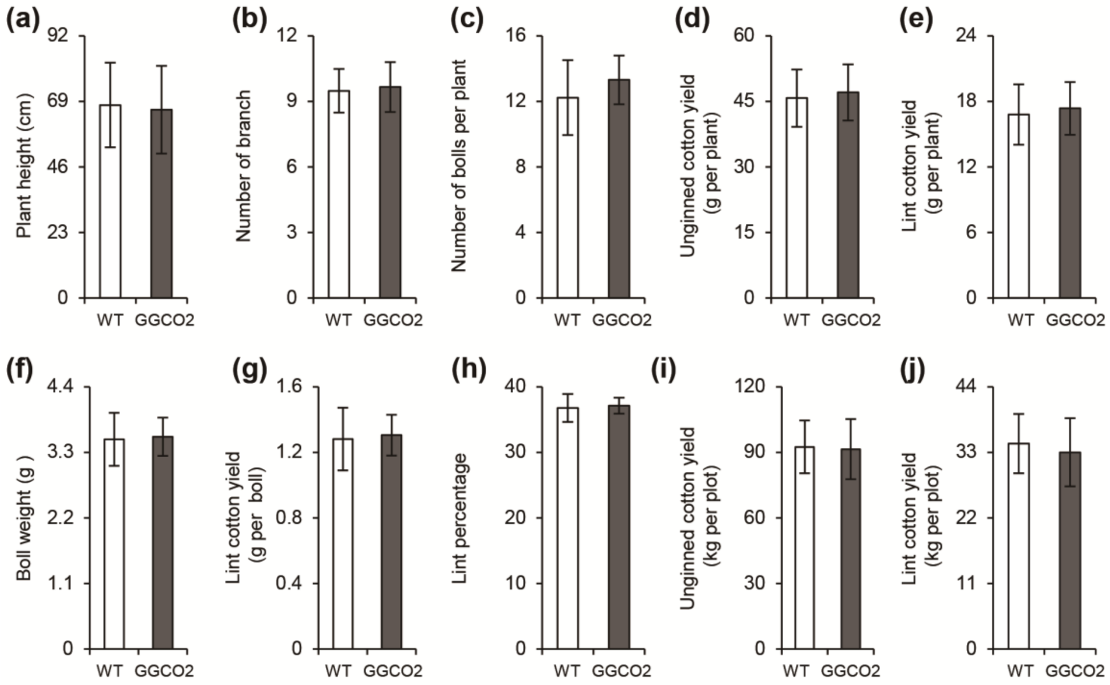

Figure S8 Agronomic traits of GGCO2 plants in normal field conditions in Langfang, Hebei province in 2015. No significant difference in agronomic traits were observed between the GGCO2 and wild-type cotton plants; traits monitored included plant height (**a**), number of branches (**b**), number of bolls per plant (**c**), unginned cotton yield per plant (**d**), lint cotton yield per plant (e), boll weight (f), lint cotton yield per boll (g), lint percentage (h), unginned cotton yield per plot (i), and lint cotton yield per plot (j). The significance of differences between the GGCO2 and wild-type cotton plants was assessed with Student's *t*-tests.

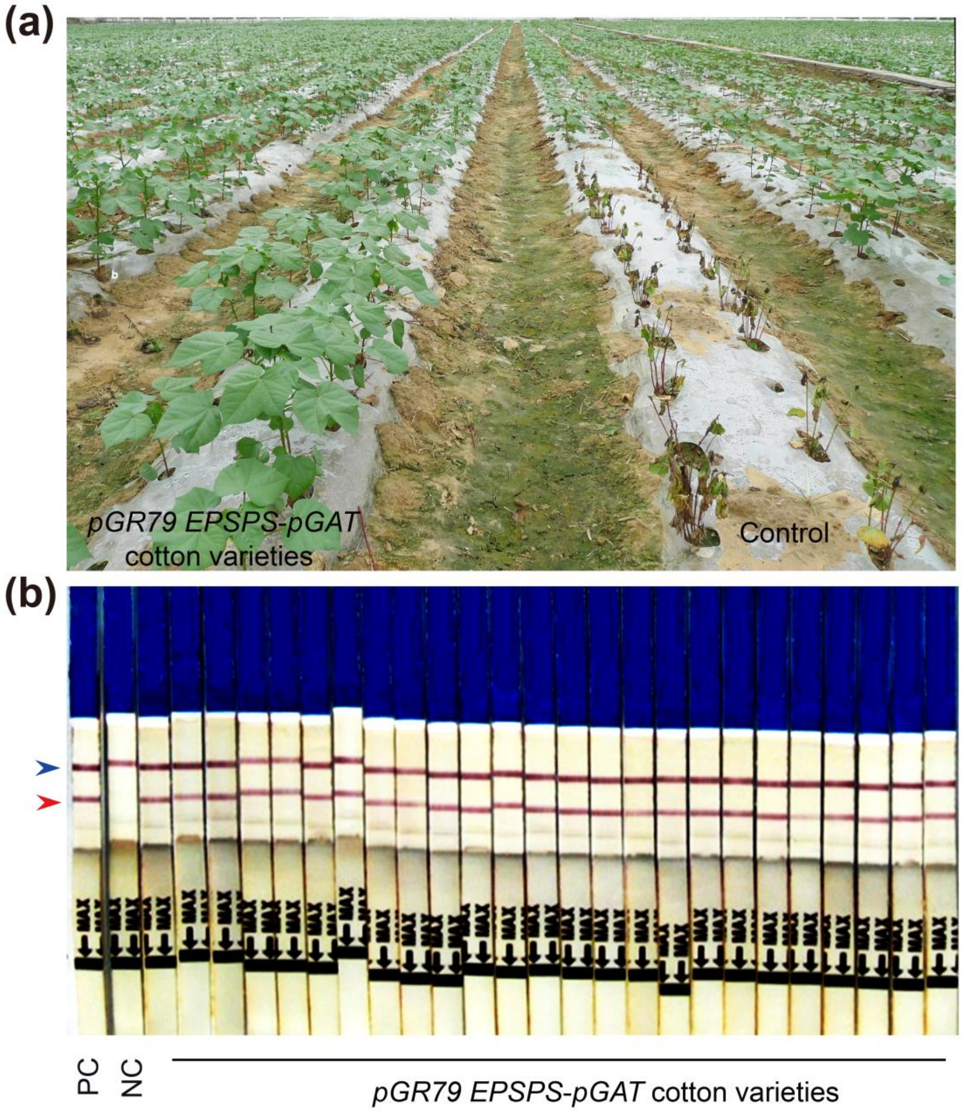

Figure S9 ImmunoStrip analysis of of the *pGR79 EPSPS-pGAT* cotton varieties. (a) A views of the breeding GR cotton varieties. Seeds from 19 independent varieties were bulked, and 100 g of seeds were sown in 30 cm × 60 cm trays. Four-week-old seedlings were sprayed glyphosate at a rate of 900 g a.e.ha^{-1}. (b) ImmunoStrip genotyping of the *pGR79-pGAT* varieties. The red arrow indicates pGR79 EPSPS capture lines. The blue arrow indicates the control line (anti-mouse lg G). Total protein extractions from 6-week-old cotton leaves were analyzed by immunoblotting using anti-GR79 EPSPS antibodies. PC, positive control. NC, negative control.

Table S1 GR varieties developed from hybridizations with GGCO2.

GR varieties	Parental varieties	Planting countries	Phenotype	
			Glyphosate	Bollworm
GSR-Yinmian1	Yinmian1	China	R	R
GSR-Lu15	Lu15	China	R	R
GSR-Yinmian8	Yinmian8	China	R	R
GSR-p53	p53	China	R	R
GSR-p30	p30	China	R	R
GSR-HND	HND	China	R	S
GSR-W98	W98	China	R	R
GSR-Yinrui361	Yinrui361	China	R	R
GSR-Chusong	Chusong	China	R	R
GSR-XiangS26	XiangS26	China	R	R
GSR-Zhong35	Zhong35	China	R	R
GSR-OD02	OD02	China	R	R
GSR-730	730	Australia	R	S
GSR-71	71	Australia	R	S
GSR-777	777	Pakistan	R	R
GSR-555	555	Pakistan	R	R
GSR-142	142	Pakistan	R	R
GSR-HY34	HY34	India	R	R
GSR-HY21	HY21	India	R	R

Table S2. Primers used in this study.

Primers for RT-PCR and qRT-PCR		
	**Forward**	**Reverse**
GR79 EPSPS	TGGGCTACCATTCCTACCAG	GCAACGTTAGTGACCCGAAT
GAT	TTTGAGGGGTGCTTTTCATC	GTGAAGAACCTGCCTTCTGC
GhActin7	TTCTGGGGATGGAGTCAGTC	TCGAGTGCCACATAAGCAAG
NtActin9	CTATTCTCCGC TTTGGACTTGGCA	AGGA CCTCAGGACAACGGAAACG
**Primers for probe amplification for Southern hybridization**		
	**Forward**	**Reverse**
GR79 EPSPS	GTCTCCAGCCAGTTCCTCTCAGGC	GTACTCAACGTGGATGCCGAACTTCTG
GAT	GATGTGAACCCTATTAACGCCGAA GAC	ATGAGGTCCCACAGGAGGAGTGTC
**Primers for GGCO transgenic cotton genotyping/screening**		
	**Forward**	**Reverse**
GR79 EPSPS	CCCTGGAGCAAGGCTACGGAGTAC	GTGGTCCCCACTGGCTGGCCTGGGT
GAT	GATGTGAACCCTATTAACGCCGA	AATTCTCTTATACATCAAAATATGA
NPTII	ATGATTGAACAAGATGGATTGCAC	TCAGAAGAACTCGTCAAGAAGGCG
GhActin7	GGATGGAGTCAGTCACACAGTT	ATTGATGAGCTGCTTTTCGCAG
P1	AAGATAACTTTTACCTGCCAATGCT	
P2	GCGGCTGAGTGGCTCCTTC	
P3	CAACTCTCTGAGGGCCAGGCGGTGAAG	
P4	GGTTTCTTTTTGGACACCCTCTACG	
